# Gibberellins Inhibit Flavonoid Biosynthesis and Promote Nitrogen Metabolism in *Medicago truncatula*

**DOI:** 10.3390/ijms22179291

**Published:** 2021-08-27

**Authors:** Hao Sun, Huiting Cui, Jiaju Zhang, Junmei Kang, Zhen Wang, Mingna Li, Fengyan Yi, Qingchuan Yang, Ruicai Long

**Affiliations:** 1 Institute of Animal Sciences, Chinese Academy of Agricultural Sciences, Beijing 100193, China; sunhao921023@163.com (H.S.); cuihting@163.com (H.C.); zhangjjzeus@163.com (J.Z.); kangjunmei@caas.cn (J.K.); wangzhen@caas.cn (Z.W.); limingna@caas.cn (M.L.); yangqingchuan@caas.cn (Q.Y.); 2 Key Laboratory of Animal Nutrition and Feed Science in South China, Ministry of Agriculture and Rural Affairs, Guangdong Key Laboratory of Animal Breeding and Nutrition, Institute of Animal Science, Guangdong Academy of Agricultural Sciences, Guangzhou 510640, China; 3 Inner Mongolia Academy of Agricultural and Animal Husbandry Sciences, Hohhot 010000, China; yifengyanonly88@126.com

**Keywords:** *Medicago truncatula*, gibberellin, proteomics, metabolomics, flavonoid biosynthesis, nitrogen metabolism

## Abstract

Bioactive gibberellic acids (GAs) are diterpenoid plant hormones that are biosynthesized through complex pathways and control various aspects of growth and development. Although GA biosynthesis has been intensively studied, the downstream metabolic pathways regulated by GAs have remained largely unexplored. We investigated Tnt1 retrotransposon insertion mutant lines of *Medicago truncatula* with a dwarf phenotype by forward and reverse genetics screening and phylogenetic, molecular, biochemical, proteomic and metabolomic analyses. Three Tnt1 retrotransposon insertion mutant lines of the gibberellin 3-beta-dioxygenase 1 gene (*GA3ox1*) with a dwarf phenotype were identified, in which the synthesis of GAs (GA_3_ and GA_4_) was inhibited. Phenotypic analysis revealed that plant height, root and petiole length of *ga3ox1* mutants were shorter than those of the wild type (*Medicago truncatula* ecotype R108). Leaf size was also much smaller in *ga3ox1* mutants than that in wild-type R108, which is probably due to cell-size diminution instead of a decrease in cell number. Proteomic and metabolomic analyses of *ga3ox1*/R108 leaves revealed that in the *ga3ox1* mutant, flavonoid isoflavonoid biosynthesis was significantly up-regulated, while nitrogen metabolism was down-regulated. Additionally, we further demonstrated that flavonoid and isoflavonoid biosynthesis was induced by prohexadione calcium, an inhibitor of GA3ox enzyme, and inhibited by exogenous GA_3_. In contrast, nitrogen metabolism was promoted by exogenous GA_3_ but inhibited by prohexadione calcium. The results of this study further demonstrated that GAs play critical roles in positively regulating nitrogen metabolism and transport and negatively regulating flavonoid biosynthesis through GA-mediated signaling pathways in leaves.

## 1. Introduction

Plant hormones, through endogenous biosynthesis and signal transduction in plants, perform complex physiological functions at very low concentrations. Plant hormones play important roles in plant developmental and physiological processes, such as morphogenesis, growth, metabolism, etc. [1]. Gibberellic acids (GAs), a diterpenoid plant hormone, play an important role in various processes in the whole life cycle of plants, including seed germination, hypocotyl and stem elongation, leaf expansion, trichome development, flowering time, flower development and fruit development [2,3].

GA biosynthesis can be divided into three stages, which occur in three independent cell regions [3,4,5]. The first stage is the production of ent-kaurene from geranylgeranyl diphosphate (GGDP) catalyzed by ent-copalyl diphosphate synthase (CPS) and ent-kaurene synthase (KS). It has been shown that these two key enzymes catalyzing this reaction are located in the plastid, indicating that the first stage occurs in the plastid [6,7]. The second stage mainly consists of two steps. First, ent-kaurene oxidase (KO) catalyzes the formation of ent-kaurenoic acid, which occurs mainly in the plastid membrane. Then, ent-kaurenoic acid is catalyzed to GA_12_ by ent-kaurenoic acid oxidase (KAO) in the endoplasmic reticulum [7]. In the third and last stage, which occurs in the nucleus and cytoplasm [8], bioactive GAs (including GA_1_, GA_3_, GA_4_ and GA_7_) are synthesized from the precursor GA_12_ and catalyzed by the oxidases GA20ox and GA3ox [3]. Furthermore, as a key enzyme in GA synthesis, GA3ox1 is responsible for catalyzing GA_9_/GA_20_ to generate GA_4_/GA_1_.

Among more than 130 GAs discovered to date, both 13-OH and 13-H GAs frequently coexist in the same tissue, including GA_4_, GA_1_ (also known as 13-OH GA_4_), GA_7_ and GA_3_ (also known as 13-OH GA_7_) are common bioactive GAs in flowering plants. Although bioactivity of GA_1_ (a 13-OH GA) is lower than GA_4_ (a 13-H GA) in plants [9], the predominant bioactive form depends on plant species [10]. Furthermore, endogenous bioactive GA levels are also regulated by deactivation. Gibberellin 2-oxidase (GA2ox) encodes a 2-oxoglutarate-dependent dioxygenase (2ODD) and converts bioactive and intermediate forms of GAs to their inactive forms by 2β-hydroxylation [11]. Mutants defective in GA deactivation are taller than WT plants, including *cyp714d1/eui* of rice [12], *slender* of pea (*Psga2ox1*) [13], *cyp714a1/cyp714a2* of Arabidopsis [14] and an Arabidopsis *ga2ox* quintuple mutant [15]. In addition, CYP714B1 and CYP714B2, two cytochrome P450 genes encoding GA 13-oxidase, were previously identified to be responsible for converting GA_12_ into GA_53_ (13-OH GA_12_) in rice. Moreover, CYP714B1 and CYP714B2 play a role in fine-tuning plant growth by decreasing GA bioactivity, and that they also participate in GA homeostasis [16]. Similarly, CYP72A9, a cytochrome P450 gene encoding an active GA 13-hydroxylase, was identified to be responsible for catalyzing the conversion of 13-H GAs to the corresponding 13-OH GAs in *Arabidopsis* [17]. Further investigation demonstrated that over-expression of CYP72A9 or CYP714B1/CYP714B2 resulted in the reduction of GA_4_, whereas the level of GA_1_ was increased, and the transgene lines finally exhibited a semi-dwarfism phenotype.

GA-deficient mutants of genes involved at early (*ga1-3* mutants), intermediate (*kao1 kao2* mutants) and late-stage (*ga20ox1 ga20ox2 ga20ox3* and *ga3ox1 ga3ox2* mutants) of the GA biosynthesis pathway show typical GA-deficient phenotypes such as severe dwarfism and late flowering [18]. Over past decades, *GA3ox* has been isolated and cloned from *Arabidopsis* [19], alfalfa [20], aspen tree [21], *Medicago truncatula* [22], pea [13], pumpkin [23], rice [24], tobacco [25] and wheat [26]. Structural analysis of the protein encoded by *ga3ox* showed that it contains a Fe^2+^ ion domain and a 2-oxoglutarate-dependent dioxygenase (2ODD) conserved domain [27]. GA3ox plays a role in regulating seed germination, stem elongation, leaf development, flowering and fruiting by controlling the generation of active GAs [3,4]. The generation of bioactive GAs catalyzed by GA3ox is regulated through a complex regulatory process, involving internal hormonal homeostasis (including signal pathway, hormone level, etc.) and external environmental factors (including light, temperature and other factors) to control plant growth by influencing the transcription level and spatiotemporal expression mode of the *GA3ox* gene.

Besides GA biosynthesis and metabolism, the GA signaling transduction pathway is also tightly regulated during plant growth and development, such as root nodulation. Recently, the influence of gibberellin on the regulation of nodulation and nitrogen fixation in legumes has been emerged, which further broadened the biological function of gibberellin. Early literature demonstrated that gibberellin is required to promote nodule organogenesis; the absence of gibberellin led to the decrease of nodule number and the abnormal development of nodules [28,29]. Subsequent studies showed that gibberellin concentration at a high or low level was not conducive to the occurrence of nodules [29]. In addition, a dual role for GA during infection thread formation and nodule development was validated, suggesting that gibberellin promotes nodule organogenesis and inhibits the formulation of an infection thread [30,31]. Further analysis showed that gibberellin played a role in maintaining the size and activity of the nodule, but it could inhibit the formation of the number of nodules [32]. Furthermore, the CRISPR/Cas9-mediated deletion mutation of *MtGA2ox10* inhibited nodule formation and retarded nodule development, while over-expression of *MtGA2ox10* increased infection thread formation but inhibition of nodule development, which further demonstrated that gibberellin positively regulated the development of nodule and reduced the formation of the nodule by inhibiting the formation of an infection thread [33]. More recently, the restriction of rhizobial development and the decrease of nitrogen fixation efficiency were observed in gibberellin-deficient pea *na* mutants [34].

Although the regulatory molecular mechanism of plant morphogenesis by GAs has been partly elucidated, the role of gibberellin in regulating downstream metabolic pathways needs to be further revealed. Relevant researches have shown that the application of orthogonal analytical methods, such as two-dimensional (2D) gel electrophoresis, liquid chromatography-mass spectrometry (LC-MS) and gas chromatography-mass spectrometry (GC–MS) can not only result in more overall coverage of biological systems but can also generate complementary datasets that can be interpreted through ontological and correlation analyses [35]. In the current study, proteomics based on iTRAQ labeling and metabolomics based on high-performance liquid chromatography-mass spectrometry (HPLC-MS) of wild-type R108 and *ga3ox1* mutant leaves were carried out simultaneously to elucidate physiological change and metabolic adaption. To further investigate the potential downstream metabolic pathway of gibberellin regulation, andprovide new ideas for the exploration of new functions of gibberellin in legumes.

## 2. Results

### 2.1. Isolation and Characterization of M. truncatula ga3ox1 Mutants

**Figure 1:**
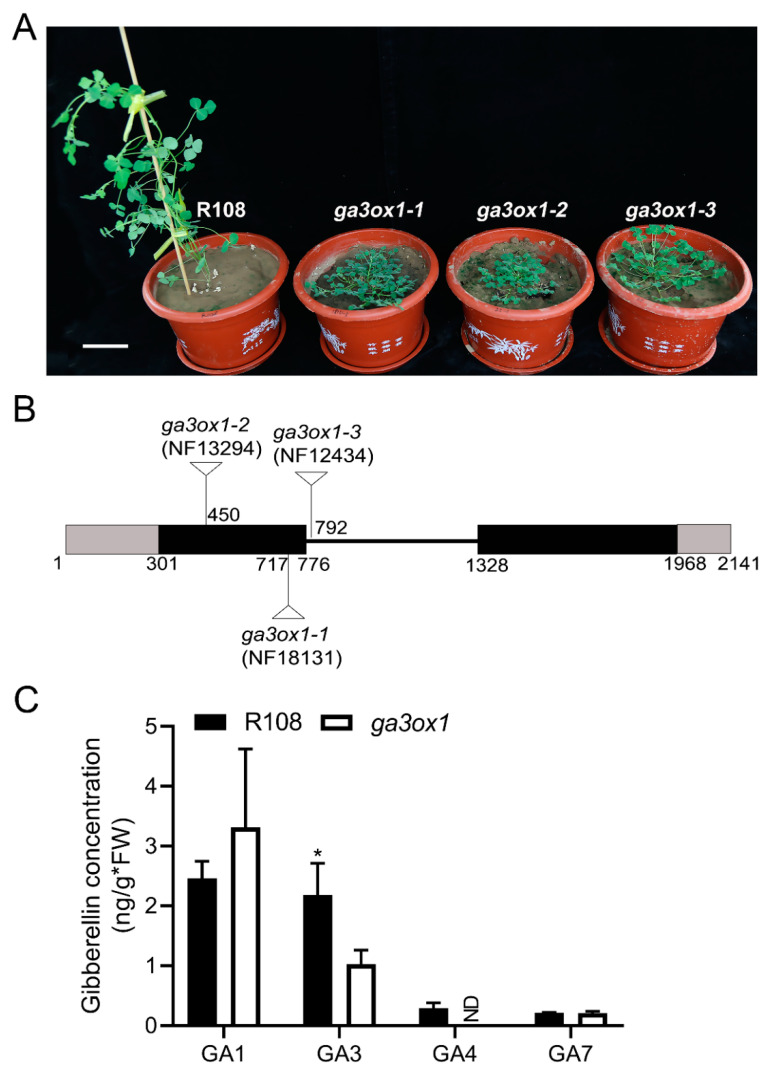
Screening and identification of *ga3ox1* mutants. (**A**) Phenotypes of *ga3ox1* null mutants. The *ga3ox1* mutant displays a dwarf phenotype. Bar = 5 cm. (**B**) Position of *Tnt1* insertions in the *GA3ox1* gene in the mutant lines *ga3ox1-1* (NF18131), *ga3ox1-2* (NF13294) and *ga3ox1-3* (NF12434). Gray boxes indicate the 5′UTR and 3′UTR. Dark boxes indicate exons, and the line indicates the intron. (**C**) Determination of the content of bioactive GAs content based on HPLC-MS analysis. Three biological replicates were analyzed. Error bars indicate SD. * *p* < 0.05 by Duncan test. NA: not available.

Three Tnt1 insertion mutant lines of *MtGA3ox1* (NF13294, NF18131 and NF12434) were screened from an *M. truncatula* Tnt1 mutant population from the Noble Research Institute (Figure 1A). The TAIL-PCR results showed that the Tnt1 insertion in NF18131 was located in the first exon of *MtGA3ox1* at 416 bp downstream of the ATG start codon (Figure 1B). In addition, the Tnt1 insertions in NF13294 and NF12434 were confirmed to be located in the first exon of *MtGA3ox1* at 149 bp downstream of the ATG start codon and the intron of *MtGA3ox1* at 16 bp downstream of the first exon, respectively (Figure 1B). In order to confirm the Tnt1 insertion, RT-PCR detection was performed at the DNA and cDNA levels. Due to Tnt1 insertion, RT-PCR could not detect the full-length *GA3ox1* transcript in lines NF13294 (*ga3ox1-2*), NF18131 (*ga3ox1-1*) and NF12434 (*ga3ox1-3*), indicating that they are null mutants (Supplementary Materials Figure S1A). Although *Mtga3ox* mutant has been recently characterized in other mutant lines of *M. truncatula* [26], the mutant lines used in our experiment were identified by forward genetics combined with reverse genetics. Since the subsequent experiments were carried out with *ga3ox1-1*, we donated *ga3ox1-1* as *ga3ox1.* In a phylogenetic analysis of MtGA3ox1, based on the entire protein sequences, MtGA3ox1 was clustered with a group of proteins that have been identified as the GA3ox family of proteins (Supplementary Materials Figure S1B), which are responsible for catalyzing the reactions from GA_9_ to GA_4_, as well as from GA_20_ to GA_1_ (Supplementary Materials Figure S1C) [5]. The content of the various bioactive GAs in the wild-type R108 and *ga3ox1* mutants was determined by HPLC-MS. The results revealed that the content of GA_3_ and GA_4_ in the *ga3ox1* mutant was significantly lower than that in the R108 ecotype. Compared with wild-type R108, the content of GA_3_ in the *ga3ox1* mutant was reduced by 50%, while the content of GA_4_ was undetectable (Figure 1C). In addition, we further used HPLC/MS analysis to profile the endogenous GAs in wild-type R108 and *ga3ox1* mutants. The results showed that several GAs (GA_1_, GA_3_, GA_3_ O-beta glucoside, GA_4_, GA_5_) were greatly reduced in the *ga3ox1* mutant, whereas GA_36_ was greatly increased compared with that in wild-type R108 (Supplementary Materials Figure S1D). In addition, GA_9_ and GA_20_ maintained at a normal level. Overall, these data suggested that the loss function of MtGA3ox1 resulted in a decrease in various bioactive GAs (GA_1_, GA_3_ and GA_4_) and an increase in GA_36_.

### 2.2. Phenotypic Analysis and Physiological Changes of the ga3ox1 Mutant

**Figure 2:**
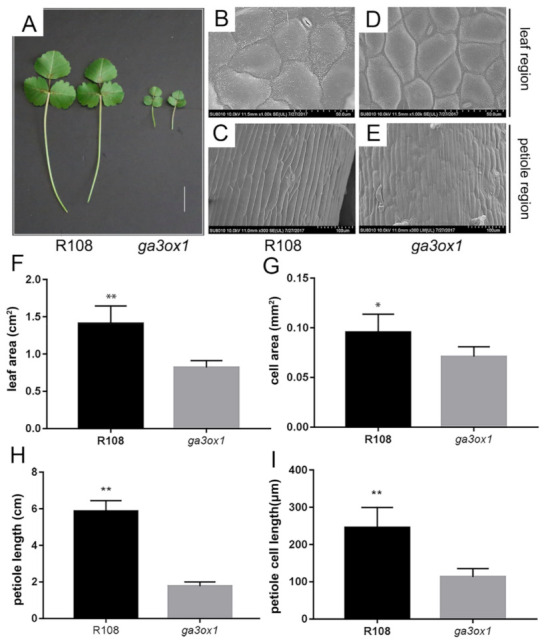
Phenotype identification and analysis of the *ga3ox1* mutant. (**A**) Phenotype of *ga3ox1* mutant leaves, petioles. Bar = 2 cm. (**B**–**E**) SEM images of leaves with a magnification of 1000× and petioles with a magnification of 300× of wild-type R108 and *ga3ox1* mutants, respectively. (**F**) Leaf area of wild-type R108 and *ga3ox1* mutants were measured with a leaf area meter. Six single leaves were randomly selected to calculate the cell area. (**G**) Leaf cell area of wild-type R108 and *ga3ox1* mutant calculated using the ImageJ software (https://imagej.nih.gov/ij/ accessed on 26 July 2021) [36]. Ten single cells were randomly selected to calculate the leaf area. (**H**) Petiole length of wild-type R108 and *ga3ox1* mutant was measured with a ruler. Six single petioles were randomly selected to calculate the petiole length. (**I**) Petiole cell length of wild-type R108 and *ga3ox1* mutant calculate using the ImageJ software (https://imagej.nih.gov/ij/ accessed on 26 July 2021) [36], respectively. Ten single cells were randomly selected to calculate the petiole cell length. Error bars indicate SD. * *p* < 0.05, and ** *p* < 0.01 by Duncan test.

Compared with wild-type R108, the Tnt1 insertion mutant lines showed a dwarf phenotype, creeping growth, dark green leaves, shorter petioles and smaller leaves (Figure 2A). SEM imaging revealed that the leaf cell size and petoile cell length were significantly larger in wild-type R108 than that in *ga3ox1* mutant (Figure 2B–E), which was consistent with leaf area and petiole length (Figure 2F–I). In order to establish the relationship between leaf color and chlorophyll content, the chlorophyll content of *ga3ox1* mutant and wild-type R108 was determined. The results showed that the content of chlorophyll a (Ca), chlorophyll b (Cb) and carotenoid (Cx.c) in the *ga3ox1* mutant leaves was significantly higher than those in wild-type R108 (Supplementary Materials Figure S2A,B). Similarly, the seed coat displayed a darker color in the *ga3ox1* mutant (Supplementary Materials Figure S2C). The content of flavonoids in seeds, where they are involved in seed coat pigmentation, was determined, and the content of flavonoids was consistently higher in the *ga3ox1* mutant (Supplementary Materials Figure S2C,D).

To further investigate the relationship between the MtGA3ox1 and dwarf phenotype, we cloned the *MtGA3ox1* coding region and the complete gene and constructed the complementary vector *pGA3ox1: GA3ox1/ga3ox1* and the overexpression vector *p35S: GA3ox1/ga3ox1* with GUS fusion tag. Phenotypic characterization revealed that ectopic-expression of *MtGA3ox1* in the *ga3ox1* mutant restored the upright phenotype, as well as leaf size, petiole length and seed coat color to a similar phenotype as that of wild-type R108 (Supplementary Materials Figure S3A,B), which further demonstrated that the *ga3ox1* mutant phenotype is indeed caused by loss of function of MtGA3ox1.

### 2.3. Loss of Function of MtGA3ox1 Inhibits Plant Morphogenesis

**Figure 3:**
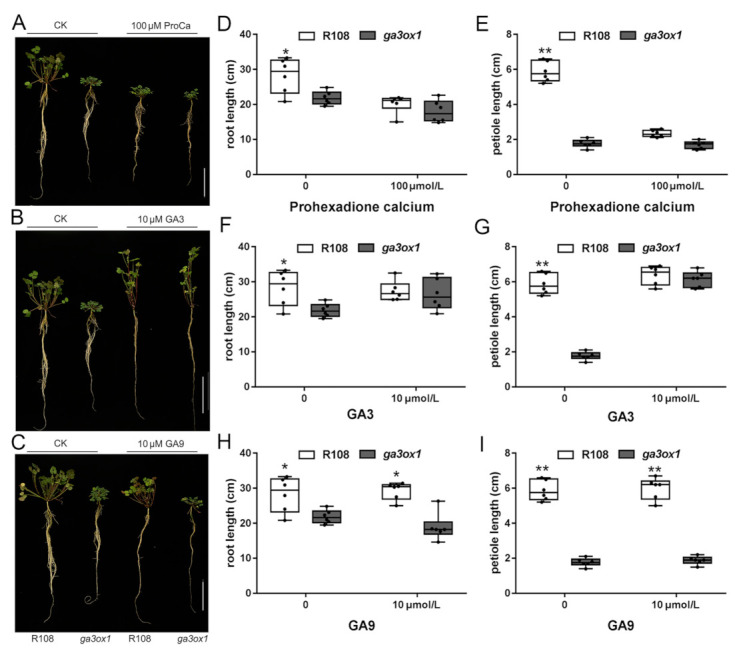
The dwarfism of *ga3ox1* mutants resulted from bioactive GA deficiency. (**A**–**C**) Phenotype identification of wild-type R108 and *ga3ox1* mutants treated with GAs and MtGA3ox1 enzyme inhibitors; note: control group (R108, *ga3ox1*) and treatment group (R108, *ga3ox1*) from left to right. (**D**–**I**) Root length and petiole length of wild-type R108 and *ga3ox1* mutants treated with (**D**,**E**) 100 μM prohexadione calcium, (**F**,**G**) 10 μM GA_3_, (**H**,**I**) 10 μM GA_9_. Six biological replicates were analyzed, and error bar indicates SD. Bars = 10 cm. * *p* < 0.05, and ** *p* < 0.01 by Duncan test.

To further illustrate that the dwarf phenotype resulted from the Tnt1 insertion into *MtGA3ox1*, prohexadione calcium, a known inhibitor of GA3ox enzymes [37], was used for exogenous treatment of *M. truncatula* seedlings. The results showed that the phenotype of the R108 ecotype was consistent with the dwarf phenotype under 100 µM prohexadione calcium (Figure 3A,D,E). Furthermore, GA_3_ is one of the few bioactive GAs, as well as one of the products of the catalytic reaction of MtGA3ox1. Accordingly, GA_9_, which is one of the precursors of the biosynthesis of GA_3_ and GA_4_, has no bioactivity. To further understand the relationship between GA deficiency and the dwarf phenotype, *M. truncatula* seedlings were separately treated with GA_3_, GA_9_ and prohexadione calcium. The results revealed that GA_3_ could effectively restore the dwarf phenotype of the *ga3ox1* mutant to the normal state consistent with wild-type R108 (Figure 3B,F,G), while GA_9_ treatment had no effect on the *ga3ox1* mutant and wild-type R108 (Figure 3C,H,I). These results indicated that the dwarf phenotype likely resulted from deficiency of bioactive GAs, while exogenous GA_9_ could not be catalyzed to generate bioactive GAs due to the Tnt1 insertion mutation of *MtGA3ox1*.

As mentioned above, due to the lack of GAs, leaf and petiole lengths become smaller. Therefore, four bioactive GAs (GA_1_, GA_3_, GA_4_ and GA_7_) were used to investigate the relationship between bioactive GAs and the dwarf phenotype. Remarkably, to a certain extent, four bioactive GAs could consistently restore the dwarf phenotype of the *ga3ox1* mutant to the wild-type phenotype (Supplementary Materials Figure S4A). In addition, the cell number and cell area of the *ga3ox1* mutant were restored to the normal level, which was consistent with those of wild-type R108 (Supplementary Materials Figure S4B–E). However, although treatment with GAs did not significantly change cell size, it significantly increased the hypocotyl length in wild-type R108 (Supplementary Materials Figure S4A).

### 2.4. Based on iTRAQ Labeling Proteomic Analysis and HPLC-MS Metabolomic Analysis

**Figure 4:**
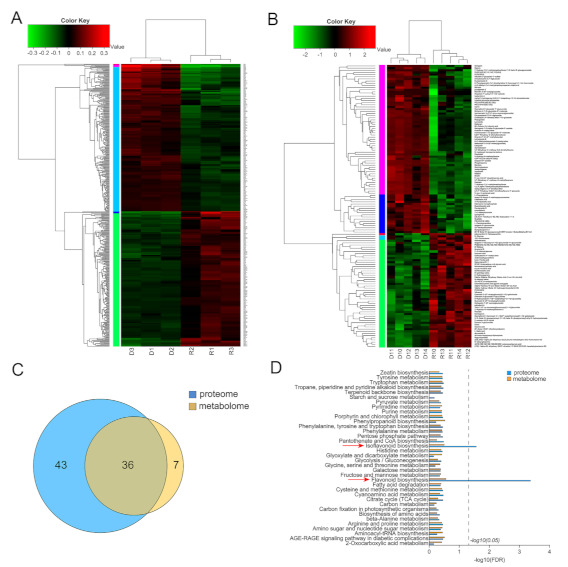
Proteomic and metabolomic analysis of wild-type R108 and *ga3ox1* mutant leaves. (**A**) The heatmap of differentially enriched proteins (DEPs) identified by proteomics. (**B**) The heatmap of differential metabolites identified by metabolomics. (**C**) Venn diagram of KEGG pathways enriched by differential proteins and metabolites. (**D**) The histogram of KEGG enrichment of differential proteins/metabolites.

To further investigate dynamic changes resulting from GA deficiency in *ga3ox1* mutant, proteomics based on iTRAQ labeling and metabolomics based on HPLC-MS analysis were performed on *ga3ox1*/R108 tissues. In this study, a total of 456 significantly differentially enriched proteins (DEPs) (screening criteria: *p* < 0.05 & (FC < 0.83 or FC > 1.20)) were identified, including 238 up-regulated and 218 down-regulated enriched proteins (Figure 4A, Supplementary Materials Table S1). Among them, the up-regulated DEPs were mainly involved in flavonoid, isoflavone and lignin biosynthesis (Supplementary Materials Figure S5A, Supplementary Materials Table S2), while the down-regulated DEPs were mainly involved in oxidative phosphorylation, nitrogen metabolism, photosynthesis and other metabolic pathways (Supplementary Materials Figure S5B, Supplementary Materials Table S2).

In addition, a total of 131 differential metabolites were screened and identified by using the NIST spectral library attached to GC/MS and coupled to the multi-dimensional analysis of OPLS-DA with the single-dimensional analysis of Student’s *t*-test (VIP > 1.5, *p* < 0.05), including 53 up-regulated differential metabolites and 78 down-regulated ones (Figure 4B, Supplementary Materials Table S3). Additionally, differential metabolites were assigned to seven biological processes, including glycine, serine and threonine metabolism, starch and sucrose metabolism, ABC transporters, carbon metabolism, aminoacyl-tRNA biosynthesis, isoflavonoid biosynthesis and phenylpropanoid biosynthesis (Supplementary Materials Figure S5C, Supplementary Materials Table S2).

By comparing the metabolic pathways identified to be involved by DEPs based on proteomics and those identified to be involved by differential metabolites based on metabolomics, it was found that DEPs were annotated to 79 metabolic pathways, while differentially enriched metabolites were annotated to 43 metabolic pathways (Figure 4C). Among them, 36 metabolic pathways were consistently enriched between proteomics and metabolomics, including phenylpropanoids synthesis, carbon fixation in photosynthetic organs, flavonoid synthesis, lignin synthesis, isoflavone synthesis, glycolysis and glyconeogenesis. Further KEGG (Kyoto Encyclopedia of Genes and Genomes) enrichment analysis of proteomics and metabolomics metabolic pathways revealed that the flavonoid and isoflavonoid biosynthesis pathways were significantly enriched when the screening condition was FDR < 0.5 (Figure 4D), which indicated that there were dynamic changes in flavonoid and isoflavone synthesis between wild-type R108 and *ga3ox1* mutants.

### 2.5. GAs Inhibited Flavonoid and Isoflavonoid Biosynthesis

**Figure 5:**
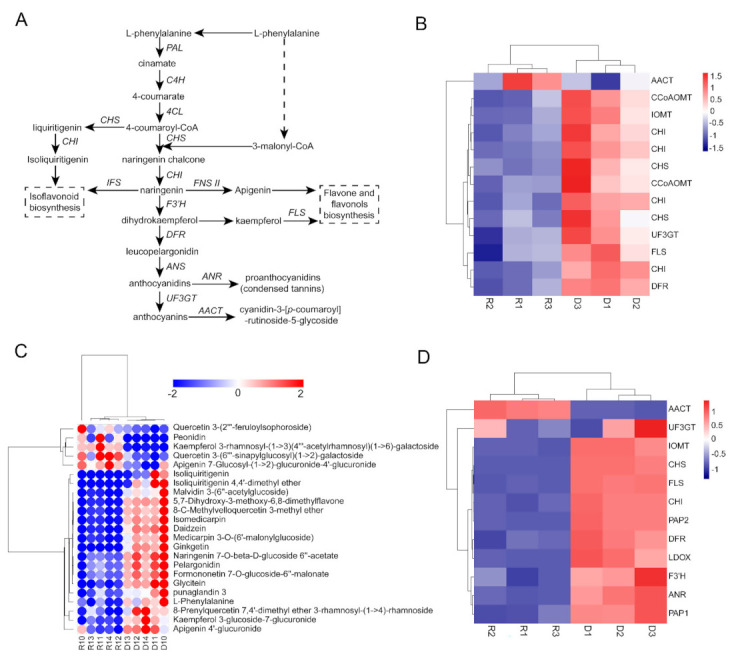
GAs inhibited the flavonoid and isoflavonoid biosynthetic pathways. (**A**) Anthocyanin biosynthesis pathway. (**B**) The heatmap of DEPs involved in flavonoid and isoflavonoid biosynthesis. (**C**) The heatmap of differential metabolites associated with flavonoid and isoflavonoid. (**D**) Transcription levels of flavonoid biosynthesis genes in wild-type R108 and *ga3ox1* mutant. The heatmap was performed using the OmicShare tools, a free online platform for data analysis (http://www.omicshare.com/tools, 8 May 2020). According to the color scale on the right of Figure, each colorized cell represents the averaged spot quantity, and the color scale indicates the fold change in the mRNA expression level.

To further establish the relationship between the GA and anthocyanin biosynthesis pathways, several key enzymes identified in the proteomic analysis as involved in anthocyanin biosynthesis pathways were selected to investigate the abundance of their corresponding mRNA and protein. The results showed that the mRNA transcript and protein of the key enzymes involved in the anthocyanin biosynthesis pathway (Figure 5A) were present at higher abundance in the *ga3ox1* mutant. Meanwhile, the abundance of mRNA transcript and protein of AACT (Anthocyanin 5-aromatic acyltransferase), a protein related to the acetylation of anthocyanin, was lower in the *ga3ox1* mutant (Figure 5B,C). In addition, the transcript level of *PAP1*(production of anthocyanin pigment (1) and *PAP2* (production of anthocyanin pigment (2), which regulate the anthocyanin synthesis pathway, were significantly up-regulated in *ga3ox1* mutant (Figure 5C).

**Figure 6:**
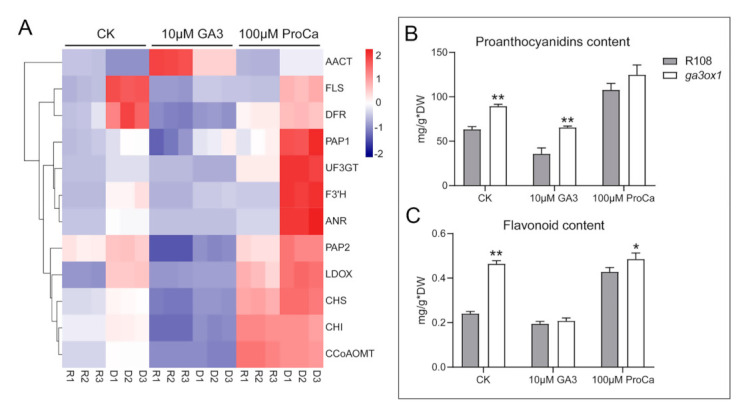
Treatment with exogenous GA_3_ inhibited flavonoid and isoflavonoid biosynthesis pathways, while treatment with prohexadione calcium promoted these processes. (**A**) Transcriptional abundance of flavonoid biosynthesis genes after treatment with exogenous GA_3_ and prohexadione calcium. The heatmap was built using the OmicShare tools, a free online platform for data analysis (http://www.omicshare.com/tools, 9 May 2020). According to the color scale on the right of the Figure, each colorized cell represents the averaged spot quantity, and the color scale indicates fold change of the mRNA expression levels. (**B**,**C**) Flavonoid content and proanthocyanin content of wild-type R108 and *ga3ox1* mutant leaves were determined by spectrophotometer. Three biological replicates were analyzed, and error bars denote the SD. DW: dry weight. * *p* < 0.05, and ** *p* < 0.01 by Duncan test.

To further confirm the higher accumulation of flavonoid and anthocyanin due to GA deficiency, the gene expression levels of key naringenin downstream enzymes were analyzed after treatment with exogenous GA_3_ and prohexadione calcium (Figure 6A). The results showed that the transcript abundance of key enzymes involved in the anthocyanin synthesis pathway was significantly down-regulated after GA_3_ treatment, and the AACT involved in the acetylation of anthocyanin was significantly up-regulated (Figure 6A). Moreover, the analysis of flavonoid and proanthocyanin contents revealed that the levels of flavonoids and proanthocyanin were significantly increased in the *ga3ox1* mutant (Figure 6B,C). Furthermore, the transcript abundance of *PAP1* and *PAP2*, as well as the content of flavonoids and proanthocyanin, were also significantly decreased by treatment with exogenous GA_3_ but increased by treatment with exogenous prohexadione calcium treatment, which further demonstrated the inhibitory effect of GA on the anthocyanin synthesis pathway. Likewise, the mRNA abundance of key enzymes associated with flavonoid and isoflavonoid biosynthesis, as well as proanthocyanin content, decreased in complementary lines *p35S:GA3ox1/ga3ox1* and *pGA3ox1:GA3ox1/ga3ox1*, which further confirmed that GA biosynthesis inhibits flavonoid and isoflavonoid biosynthesis (Supplementary Materials Figure S6A–I).

**Figure 7:**
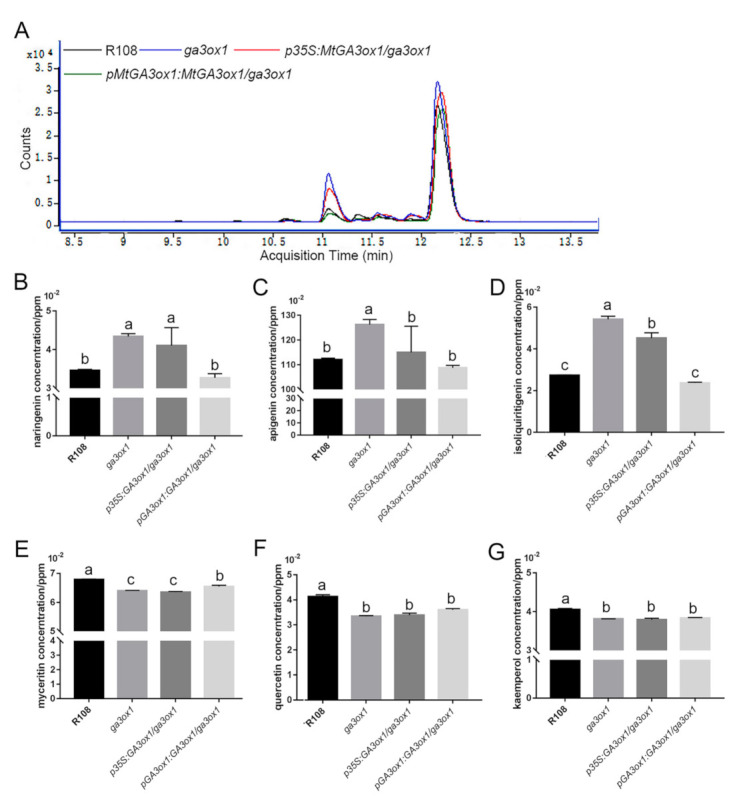
HPLC chromatograms and absorbance spectra of flavonoid extracts from leaves. (**A**) Chromatograms of leaf extracts from 1-month-old wild-type R108 (black line), *ga3ox1* mutant (blue line) and *p35S:MtGA3ox1/ga3ox1* (red line), *pMtGA3ox1: MtGA3ox1/ga3ox1*(green line) leaves. (**B**–**G**) The absolute content of naringenin, apigenin, isoliquiritigenin, myceritin, quercetin and kaemferol were determined based on the HPLC-MS analysis. Three biological replicates were analyzed, and error bars denote the SD. Different letters above the bars indicate significant difference (*p* < 0.05 by Duncan test).

To further demonstrate the function of GA3ox1 in flavonoid metabolization, the relative and absolute abundance of the main flavonoid metabolites were analyzed by HPLC/MS. The identity of the flavonoids was established using commercially available standards run under the same conditions and comparing retention times and absorbance spectra of each standard against each of the separated peaks (Supplementary Materials Figure S7). Compared with the wild type, we detected that the mutant leaves produced a higher abundance of flavonoid metabolites (Figure 7A). We further detected naringenin, dihydroquercetin, apigenin, quercetin, liquiritigenin, isoliquiritigenin, kaempferol, myricetin and dihydromyricetin as the main flavonoid metabolites. The levels of the identified flavonoids, especially naringenin and apigenin, were significantly increased in the extracts of *ga3ox1* mutant leaves (Figure 7B–D) but restored to normal levels in complementary lines *pGA3ox1:GA3ox1/ga3ox1* (Figure 7B–D). Meanwhile, the abundance of quercetin, myricetin and kaempferol, part of the downstream products of naringenin, were significantly reduced in the extracts of *ga3ox1* mutant leaves but slightly increased in complementary lines of *p35S:GA3ox1/ga3ox1* and *pGA3ox1:GA3ox1/ga3ox1* (Figure 7E–G).

### 2.6. The Effect of GA-Deficiency on Nitrogen Metabolism and Transport

**Figure 8:**
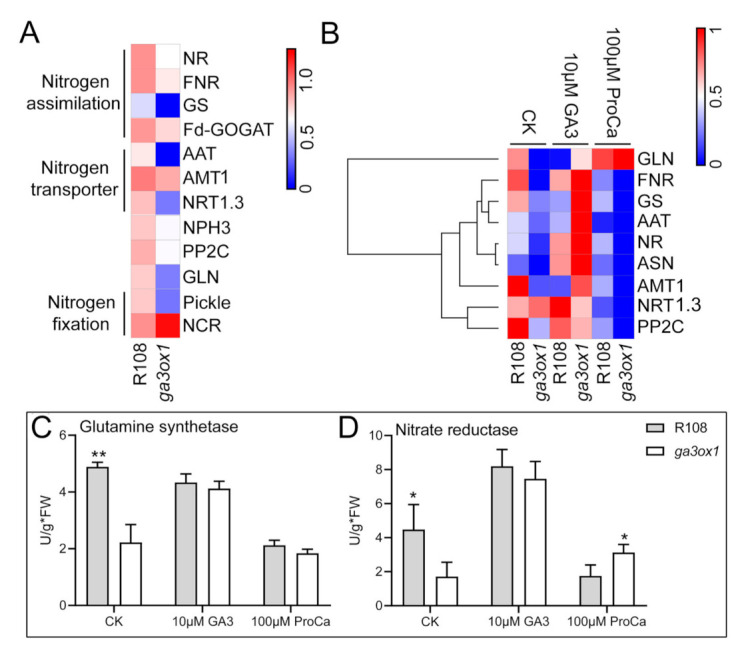
GAs induced the expression of genes related to nitrogen metabolism and positively regulated genes involved in nitrogen transport. (**A**) The heat map of DEPs involved in nitrogen metabolism and transport. (**B**) The heat map of transcriptional expression levels of differentially expressed genes related to nitrogen metabolism and transport under control, GA_3_ treatment and prohexadione calcium treatment. (**C**,**D**) Determination of glutamine synthase activity and nitrate reductase activity in 1-month-old wild-type R108 and *ga3ox1* mutant leaves under control, GA treatment and prohexadione calcium treatment. The heatmap was built using the OmicShare tools, a free online platform for data analysis (http://www.omicshare.com/tools, 19 May 2020). According to the color scale on the right of the Figure, each colorized cell represents the averaged spot quantity, and the color scale indicates fold change of the mRNA expression levels. * *p* < 0.05, and ** *p* < 0.01 by Duncan test.

Based on the iTRAQ labeling proteomic analysis, a number of DEPs associated with nitrogen assimilation (including NIR, GS, GLN and FNR), as well as DEPs associated with nitrogen transport (including NRT1.3, AAT1 and AMT1) and NPH3 involved in signal transduction of nitrogen metabolism were identified and found to be down-regulated in *ga3ox1* mutant (Figure 8A).

Although we have characterized the expression levels of several key enzymes involved in nitrogen fixation and transport, it was not sufficient evidence to explain the relationship between GAs and nitrogen metabolism. To further investigate the effect of GAs on nitrogen fixation and transport, the expression levels of DEPs associated with nitrogen metabolism and nitrogen transport were analyzed. qRT-PCR analysis was performed to confirm the abundance of mRNAs encoding NO_3_^−^ assimilation enzymes (for example, NR and FNR), amino acid transporter (AAT) and NO_3_^−^ transporters (for example, NRT1.3) in leaves. The results of the analysis revealed that the abundance of the corresponding mRNA was significantly down-regulated in *ga3ox1* mutants (Figure 8B) and by treatment with exogenous prohexadione calcium, whereas treatment with GA_3_ rescued it (Figure 8B). Meanwhile, the abundance of these mRNAs was restored in the complementary lines *pGA3ox1:GA3ox1/ga3ox1* (Supplementary Materials Figure S8A–D). Moreover, the activities of key nitrogen assimilation enzymes, such as glutamine synthase (NH_4_^+^ assimilation) [38] and nitrate reductase (NO_3_^−^ assimilation), were consistently higher in wild-type R108 than in the *ga3ox1* mutant. Similarly, the corresponding enzymatic activities were relatively enhanced in complementary lines *p35S:GA3ox1/ga3ox1* and *pGA3ox1:GA3ox1/ga3ox1* (Supplementary Materials Figure S8E,F). Notably, the activities of key nitrogen assimilation enzymes, such as glutamine synthase (NH_4_^+^ assimilation) and nitrate reductase (NO_3_^−^ assimilation), were enhanced by treatment with exogenous GA_3_ but decreased by treatment with exogenous prohexadione calcium, which had a similar trend with protein and transcript levels in the *ga3ox1* mutant (Figure 8C,D).

## 3. Discussion

### 3.1. GAs Participated in the Regulation of Flavonoid and Isoflavonoid Biosynthesis

Flavonoids are a kind of secondary polyphenolic metabolites produced by the synthesis of phenylpropanoid, which are widely found in plants, mainly including bioflavonoids, flavonols, isoflavonoids and anthocyanins. Flavonoids are essential for plant structure and function, as well as plant microbial interactions, including symbiotic signaling in root nodule symbiosis (RNS) and the genesis and development of nodule organs [39,40,41]. Anthocyanins, the key molecules of pigmentation and signal transduction, has been reported to be involved in attracting pollinators, inhibiting herbivores or pathogens, reducing the damage of reactive oxygen species and protecting the roots of legumes from ultraviolet rays [42]. Since anthocyanins are involved in various important functions, the synthesis of anthocyanins and related regulatory mechanisms have attracted extensive attention.

Several studies have shown that the biosynthesis of anthocyanins in plants is affected by external environmental factors (such as light [43], low phosphorus stress, cold stress) [44], as well as plant hormones, including CKs, ethylene, JAs and GA [45,46,47,48,49,50]. It has been shown that sucrose induced dihydroflavonol 4-reductase (DFR) and PAP1 transcription, but this process was inhibited by treatment with exogenous GA_3_, which indicated that GA_3_ is an antagonist of sucrose-induced anthocyanin biosynthesis [45]. In addition, previous studies have shown that GAs have tissue specificity in regulating anthocyanin biosynthesis, which is manifested in promoting anthocyanin biosynthesis in flowers and inhibiting anthocyanin biosynthesis in other tissues. In this study, protein and mRNA abundance of key enzymes associated with flavonoid and isoflavonoid biosynthesis, as well as differential metabolites identified by leaf metabolomics, were found consistently increased in the *ga3ox1* mutant, which indicated that these biological processes were facilitated by the absence of GAs (Supplementary Materials Figure S5A). Remarkably, we noticed that flavonoid and isoflavonoid biosynthesis, as well as flavonoid content, were restored to normal level by treatment with exogenous GA_3_ but further promoted by treatment with exogenous prohexadione calcium (Figure 6A–C). However, the abundance of protein and transcript of AACT, an enzyme associated with anthocyanin acetylation, was lower in the *ga3ox1* mutant compared with wild-type R108 (Figure 5B,C), which is consistent with previous studies [45]. These results further demonstrated that GAs have a negative effect on flavonoid and isoflavonoid biosynthesis in leaves.

However, the GA-insensitive mutant *gai* is not sensitive to GA-dependent inhibition of DFR expression induced by sucrose, which indicated that GAs regulate sucrose-induced anthocyanin biosynthesis by regulating the abundance or activity of DELLA proteins [45,50,51]. The GA–DELLA pathway has also been shown to be involved in the regulation of anthocyanin accumulation induced by low temperature [48], phosphorus deficiency [52] and nitrogen deficiency [53]. In this study, based on iTRAQ labeling proteomics analysis, a GRAS family protein was identified and found to be up-regulated in the *ga3ox1* mutant, which might provide a reasonable explanation for the anthocyanin accumulation in the *ga3ox1* mutant. Previous studies have shown that DELLA protein positively regulates nitrogen-deficiency-induced accumulation of anthocyanin through direct interaction with PAP1 and enhances its transcriptional activity on the expression of the anthocyanin biosynthesis gene [53], which suggested that GAs regulates the expression of PAP1 and its downstream genes through the regulation of the activity of DELLA proteins. Noteworthy, the transcript level of PAP1 and PAP2 were significantly up-regulated (Figure 5D) and more proanthocyanin accumulated in the *ga3ox1* mutant (Figure 6C), which indicated that GAs inhibited anthocyanin biosynthesis.

Due to the loss function of MtGA3ox1, the leaves of *ga3ox1* mutant produced a more diverse range of flavonoids metabolites (Figure 7A). Furthermore, naringenin (the precursors of flavonoid biosynthesis), apigenin (the precursors of flavone and flavonol biosynthesis) as well as isoliquiritigenin (the precursors of isoflavone biosynthesis) were significantly increased in *ga3ox1* mutant leaves (Figure 7B–D), which suggested that these three biological processes have been promoted. Meanwhile, the abundance of mRNAs and protein of the cytochrome P450 family flavone synthase, which catalyze the reaction from naringenin to apigenin, were consistently up-regulated in *ga3ox1* mutant leaves. Besides naringenin and apigenin, along with the increased abundance of mRNAs and proteins of the chalcone-flavanone isomerase family protein and chalcone and stilbene synthase family protein, the levels of liquiritigenin and isoliquiritigenin were increased in *ga3ox1* mutant leaves. These findings suggested that isoflavonoid biosynthesis, as well as flavone and flavonol biosynthesis, might be positively regulated by GAs.

### 3.2. GAs Affect Seed Coat Pigmentation by Regulating Anthocyanin Synthesis

The pigmentation of flower and seed coats is caused by the deposition of a large number of flavonoids in soybean tissues. The synthesis of these compounds mainly comes from the anthocyanin biosynthesis branch of the phenylpropane pathway [54]. Thus far, genetic analysis of genes controlling natural variation of seed coat and flower color in soybean showed that at least five alleles (I, T, W1, R and O) were known to control seed coat pigmentation, and six alleles (W1, W2, W3, W4, Wm and Wp) were known to control flower color deposition [55]. In this study, two chalcone and stilbene synthase family proteins, which have been shown to be involved in the control of the distribution of anthocyanins and proanthocyanidins [56,57], were identified and found to be up-regulated in the *ga3ox1* mutant. In addition, F3′H has been proved to be co-segregated with T locus and played a key role in control specific seed coat color by controlling the types of anthocyanins and proanthocyanidins [58]. We further investigated the transcription level of CHS and F3′H and found that the expression of related genes was significantly up-regulated in the *ga3ox1* mutant. Interestingly, compared to the wild type, the seed coat displayed a darker color in the *ga3ox1* mutant (Supplementary Materials Figure S2C), which was consistent with the mRNA abundance of CHS and F3′H. Previous studies have shown that the formation of seed coat color was mainly due to the composition of anthocyanins and proanthocyanidins. Therefore, based on leaf metabolomics, we found that peonidin and pelargonidin had a higher abundance in *ga3ox1* mutants, which may be one of the reasons for the deepening of seed coat color of the *ga3ox1* mutants. Furthermore, the expression of CHS and F3′H was induced by gibberellin synthesis inhibitors, whereas it was inhibited by exogenous GA_3_, which further demonstrated that GAs might affect seed coat pigmentation by regulating anthocyanin synthesis. Phenotypic characterization also revealed that over-expression of *MtGA3ox1* in the *ga3ox1* mutant restored seed coat color to a similar phenotype as that of wild-type R108 (Supplementary Materials Figure S3A,B). Moreover, the transcription level of CHS and F3′H were down-regulated, and the mRNA abundance corresponding genes were even lower in the *ga3ox1* mutant than that in the wild type, which provided solid evidence for the effect of GAs on seed coat color by inhibiting anthocyanin synthesis.

### 3.3. GAs Is Involved in the Regulation of Nitrogen Transport and Metabolism

Nitrogen is an essential mineral nutrient in plant growth and development, and the application of nitrogen fertilizer increased crop yield, which has become an important factor in the green revolution. The root system is responsible for absorbing and metabolizing bioavailable nitrogen and transducing nitrogen signals. Therefore, roots play a crucial role in regulating plant responses to nitrogen. Relevant research has revealed that nitrogen transporters, anabolic enzymes and signal transducers are transcriptionally regulated by changes in soil nitrogen availability [59]. Furthermore, post-transcriptional modification and calcium- and phosphorylation-dependent signaling cascades are key regulators of this transcriptional response [60]. Recently, several transcription factors associated with root development and nitrogen metabolism were screened [61,62,63,64,65,66], and carbon metabolism and hormone pathways have also been proved to be involved in nitrogen metabolism [67]. More recently, an interaction network was established based on the interaction between the promoters and genes associated with nitrogen transport, nitrogen assimilation, nitrogen signaling transduction, nitrogen metabolism as well as hormone response and transcription factors, which indicated that phytohormone probably participates in nitrogen signaling transduction [68,69]. Interestingly, nitrogen-metabolism-related proteins were identified by proteomics, and, in particular, several DEPs (including NRT1.3, AAT1 and AMT1 associated with nitrogen transport, and NIR, GS, GLN and FNR associated with nitrogen assimilation, as well as NPH3 involved in nitrogen metabolism signal transduction) were down-regulated in the *ga3ox1* mutant (Figure 8A). In addition, we further found that nitrogen metabolism and nitrogen transporter were induced by treatment with GA_3_ but inhibited by treatment with exogenous prohexadione calcium (Figure 8B), which indicated that gibberellins were required for nitrogen absorption and GAs could promote nitrogen metabolism. Recent studies reported that the decrease of bioactive GAs and the accumulation of DELLA proteins inhibited nitrogen absorption [70,71], which was consistent with our data. In addition, the accumulation of DELLA proteins resulted in semi-dwarfing, which reduced the response of plant growth to nitrogen, thus reducing the absorption of nitrogen. Moreover, the abundance of DELLA proteins probably affects NO_3_^−^ uptake and nitrate reductase activity by inhibiting the GRF4-induced transcriptional activation of NO_3_^−^ metabolism genes [72]. Accordingly, we further investigated the activities of key nitrogen assimilation enzymes, including glutamine synthase (NH_4_^+^ assimilation) and nitrate reductase (NO_3_^−^ assimilation). The results showed that the corresponding enzymatic activities were lower in the *ga3ox1* mutant than those in wild-type R108, and enzymatic activities were restored in complementary lines *p35S:GA3ox1/ga3ox1* and *pGA3ox1:GA3ox1/ga3ox1* (Supplementary Materials Figure S8E,F). Furthermore, enzymatic activities associated with nitrogen assimilation were enhanced by treatment with GA_3_ but suppressed by treatment with prohexadione calcium, which suggested that nitrogen assimilation varies with dynamic changes in the GA levels. In this study, due to GA deficiency, the mRNA and protein levels of DELLA proteins were higher in the *ga3ox1* mutant compared with the wild-type R108 (Supplementary Materials Figure S9A), which reasonably explained the involvement of GAs in positively regulating nitrogen metabolism.

### 3.4. The Cross-Talk between Flavonoid Biosynthesis and Nitrogen Metabolism

The symbiotic interaction between legumes and nitrogen-fixing rhizobia bacteria provides most of the terrestrial biological nitrogen fixation. Previous studies have reported that bioflavonoids, especially flavonoids and flavones, played a critical role in legume–rhizobium symbiosis. Additionally, flavonoid-deficient lines have a near-complete loss of nodulation, while flavones-deficient lines have reduced nodulation [73]. In general, flavonoids can play a role in three distinct stages of the nodulation process. Flavonoids, as a group of inducers, promote the expression of the nod gene in the rhizosphere [74], while in the infection thread, flavonoids participate in the induction of Nod-factor biosynthesis [30]. In addition, flavonoids can also regulate auxin transport and initiate nodule primordial cell division [75,76]. Auxin transport, as well as flavonoids accumulation, are necessary for the induction of nodulation. Auxin transport is enhanced in flavonoid-deficient roots, and flavonoid deficiency hinders the inhibition of auxin transport, although NPA inhibits auxin transport [77,78]. In this study, the levels of flavonoids were higher in the *ga3ox1* mutant than those in wild-type R108. Additionally, by proteomics, auxin efflux carrier family protein, a member of auxin transporter family proteins, was identified and found to be down-regulated in *ga3ox1* mutants, which might explain the inhibitory effect of flavonoid on auxin transport [71]. Furthermore, GAs play an essential role in nodule formation and development by mediating the activities of DELLA proteins and decreasing nodule formation along with GA-deficiency [28,29]. Interestingly, in this study, the mRNA and protein levels of GRAS family transcription factor (MTR_2g097390), which was identified by proteomics, were higher in the *ga3ox1* mutant, as well as the transcript level of DELLA domain GRAS family transcription factor GAI (MTR_3g065980). DELLA proteins can be directly regulated by the nitrile specifier protein 2 (NSP2) signaling pathway and the transcription factor nuclear factor Y subunit A1 (NF-YA1), activate ERN1 transcription and promote the regulation of rhizobium infection [79]. In addition, we identified a CHD3-type chromatin-remodeling factor pickle protein (PKL) (Figure 8A), which is involved in the regulation of plant development processes, including embryonic development, seed germination, root meristem activity and hypocotyl cell elongation during skotomorphogenesis [80]. Moreover, GA_3_ inhibited H3K27me3 modification of histones associated with cell-elongation-related loci in a DELLA-mediated manner; DELLA proteins interacted with PKL and attenuated its binding ability [81]. Further analysis demonstrated that PKL plays a positive role in regulating GA signaling, and the expression of 80% of GA-responsive genes in seedlings is PKL dependent, including genes that function in cell elongation, cell division and phase transitions [82]. Therefore, PKL might display an essential role in forming indeterminate nodules. Furthermore, a Nodule Cysteine-Rich (NCR) secreted peptide (NCR) was found to be up-regulated in *ga3ox1* mutants. The gene family of NCR might be specific for galegoid legumes forming indeterminate nodules [83]; the elevated protein abundance of NCR further supported the increase of nodule number in *ga3ox1* mutants (Supplementary Figure Materials S9B). Taking plant hormones and flavonoids into account, GAs and flavonoids are important aspects in the regulation of nodule formation, respectively. Furthermore, we found an antagonistic effect between GAs and flavonoids in nodule development (Supplementary Materials Figure S9B).

The GA–DELLA system differentially regulates the mRNAs levels of the AMT1.1 and GS1.2 genes encoding proteins involved in NH_4_^+^ metabolism [38,84]. The accumulation of SLR1, a member of the DELLA protein family, reduces NH_4_^+^ uptake, whereas GA restores it by promoting protein DELLA ubiquitination and subsequent degradation [72]. In this study, due to GA deficiency, the expression of genes involved in nitrogen metabolism and transport was down-regulated and further inhibited nitrogen absorption in the *ga3ox1* mutant. Meanwhile, high carbon/nitrogen ratios induce anthocyanin pigmentation in leaves by promoting the expression of anthocyanin/flavonoid biosynthetic genes [85]. Moreover, mineral nitrogen availability reduced the isoflavonoid concentration of the soybean root system, which probably plays a part in the regulation of soybean nodule formation by available nitrogen [73]. In addition, nitrogen deficiency induced the expression of the MYB-bHLH-WD repeat protein (MBW) complex and ultimately increased the levels of anthocyanin and flavonoids in *A. thaliana* [44]. Measurement of the flavonoids content by spectrophotometry revealed that more flavonoids accumulated in the *ga3ox1* mutant, whereas flavonoid content was restored to normal levels in *pGA3ox1:GA3ox1/ga3ox1* lines. Accordingly, the abundance of pelargonidin and proanthocyanidins was increased in the *ga3ox1* mutant, which is consistent with the protein and transcription levels of key naringenin downstream enzymes. Furthermore, the cytochrome P450 family flavone synthase, identified by proteomics and found to be up-regulated in the *ga3ox1* mutant, was responsible for catalyzing naringenin to apigenin. The general flavonoid pathway regulators PAP1 and PAP2 were also up-regulated in response to nitrogen deficiency in *A. thaliana*. The transcription factor PAP1/AtMYB75 promoted the expression of genes for enzymes involved in the biosynthesis of phenylpropanoids and flavonoids, such as phenylalanine ammonialyase (PAL), chalcone synthase (CHS), chalcone isomerase (CHI) and DFR [86]. Therefore, the mRNA abundance of *PAP1* and *PAP2* were determined. Unlike previous research [44], although *PAP1* was up-regulated, *PAP2* expression was much slighter than that of *PAP1* and was even down-regulated in the *ga3ox1* mutant. In addition, DELLA proteins positively regulated nitrogen-deficiency-induced anthocyanin accumulation by directly interacting with PAP1 to enhance its transcriptional activity on anthocyanin biosynthetic gene expressions [53].

## 4. Materials and Methods

### 4.1. Plant Materials and Growth Conditions

*Medicago truncatula* cv. R108 was used as the wild type. The mutant line, NF18131, was identified from an *M. truncatula* Tnt1-insertion population based on a dwarf phenotype. A TAIL-PCR was performed to amplify and analyze flanking sequences and conduct a BLAST search against the Ensemble Plants (http://plants.ensembl.org/, accessed on 26 July 2021, version 2.0.0, EMBL-EBI, Cambridge, London) to identify the T-DNA insertion site as previously described [87,88]. Mutant and wild-type seeds were scarified with fine sandpaper and treated at 4 °C for 2 days on filter paper. Small plantlets were transferred to soil and grown in the greenhouse at 24/22 °C (day/night) temperature with 16 h light. Hydroponic culture conditions were modified from previously published work [89]. Seeds were scarified with fine sandpaper and disinfected in 20% sodium hypochlorite solution for 30 min, thoroughly washed with deionized water and subsequently germinated in a moist filter paper. Seven-day-old seedlings were then selected and transplanted to 1/2 Hoagland nutrient solution. The nutrient solutions of all seedlings were changed once per week, and the pH value was adjusted to 5.8. The temperature was maintained at 24 °C during the day and at 22 °C at night, and the relative humidity was kept at 70%.

### 4.2. TAIL-PCR

Genomic DNA was extracted according to the instructions of the Tiangen DP305 Plant Genomic DNA extraction kit (Tian-gen, Biotech Co., Ltd., Beijing, China). TAIL-PCR was performed as described previously [87]. The degenerate primers used for TAIL-PCR were AD1, AD2, AD3, AD4 and AD6 [87]. The T-DNA specific primers Tntail3, LTR4 and LTR7 (primer sequences are shown in Supplementary Materials Table S4) were used in the primary, secondary and tertiary TAIL-PCR reactions, respectively. Specific TAIL-PCR products were gel-purified, sequenced and BLAST-searched against the *Ensemble Plants* to identify the T-DNA insertion site.

### 4.3. Screening of Mutants and Plant Transformation

The mutant lines of *MtGA3ox1* (NF12434 and NF13294) were identified by searching the *Medicago truncatula* Mutant Database (https://medicago-mutant.noble.org/mutant/database.php, 6 March 2018, Noble Research Institute, Ardmore, USA). The mutants were further confirmed by PCR amplification (using primers identified from the *M. truncatula* genome sequence that spanned across the Tnt1 insertion sites). The PCR products were purified and cloned into the pEASY-T5 cloning vector (TransGen Biotech Co., Ltd., Beijing, China) and sequenced using Sanger dideoxy sequencing. The flanking sequences were BLAST-searched against the *M. truncatula* genome sequence at the NCBI database. The genomic sequence of *MtGA3ox1* was obtained from the wild-type *M. truncatula* R108 database (http://www.medicagohapmap.org/tools/r108_blastform, 18 March 2018). For *ga3ox1* mutant complementation assays, the *pMtGA3ox1:GA3ox1* and *p35S:GA3ox1* constructs were obtained using the destination vector pCAMBIA3301. For translational fusions, *MtGA3ox1* cDNA was amplified by PCR (primer sequences are shown in Supplementary Materials Table S2), first cloned into the pEASY-T5 cloning vector (TransGen Biotech Co., Ltd., Beijing, China), and subsequently in the pCAMBIA3301 (driven by the CaMV35S promoter) carrying a GUS sequence downstream of the cloning site. For wild-type R108 overexpression assays, the coding sequences of *MtGA3ox1* were obtained by RT-PCR amplification using primers MtGA3ox1-F and MtGA3ox1-R (primer sequences are shown in Supplementary Materials Table S1). The *pMtGA3ox1:GA3ox1* and *p35S:GA3ox1* constructs were obtained and used to transform the wildtype and mutant using leaf explants. PCR analysis of the regenerated plants was performed using a forward primer selected from the CaMV35S promoter (35Spromoter-F) and a reverse primer selected from MtGA3ox1 (MtGA3ox1-R1) (Supplementary Materials Table S5).

### 4.4. iTRAQ-Based Comparative Proteomic Analysis

The mature leaves of 1-month-old plants were collected and immediately frozen in liquid nitrogen. Every three individual samples were pooled for one replicate, and three biological replicates were analyzed. Total protein was isolated and purified according to a previously published study [90]. Additionally, protein concentration was measured using a Bradford assay kit (Bio-Rad Laboratories Inc., Hercules, CA, USA), and bovine serum albumin (BSA) was used as a reference protein for this experiment to generate a standard curve. Proteins were digested using the filter-aided sample preparation (FASP) method as follows [91]. The peptides of the six samples were labeled with isobaric tags from the iTRAQ Reagent-8plex Multiplex Kit (AB Sciex, LLC, Framingham, MA, USA) according to the manufacturer’s recommended procedure [92].

### 4.5. Metabolomics Analysis Based on LC-MS

Analysis of plant leaves extracts was performed by using ultra-performance liquid chromatography coupled to quadrupole-time-of-flight mass spectrometry (UPLCQ-TOF/MS) system (Waters Corporation, Milford, MA, USA), equipped with BEH C18 columns (Waters Corporation) of the following size and granulation: 100 mm × 2.1 mm, grain diameter 1.7 μm. Chromatography was performed at a flow rate of 0.40 mL/min using mixtures of two solvents: A (99.9% H_2_O, 0.1% formic acid v/v) and B (99.9% acetonitrile, 0.1% formic acid *v*/*v*). The elution steps were as follows: 0–2 min linear gradient from 5 to 20% B, 2–8 min linear gradient from 20 to 60% B, 8–12 min linear gradient from 60 to 100% B, 12–14 min isocratic at 100% B; after return to the initial conditions, the equilibration was achieved after 1 min. The settings of the TOF mass spectrometer were as follows: the signals of the mass spectrometer were collected using positive and negative ion scanning modes; electrospray capillary voltage, entrance potential and collision energy were set to 1.0 kV, 40 V and 6 eV, respectively; ion source temperature and de-solvent temperature were set to 120 and 500 °C, respectively; flow rate of dry gas was set to 900 L/h. According to the results of preliminary experiments, spectra were recorded in the targeted mode in the mass range 50–1000 *m/z*. Baseline filtering, peak identification, integration, retention time correction, peak alignment and normalization of the original data were performed by the metabolomics software progenesis QI (Waters Corporation), and a data matrix of the retention time, mass/charge ratio and peak strength was ultimately obtained. After normalization of the data matrix, it was imported into the SIMCA-P 14.0 software (Umetrics, Umea, Sweden); the unsupervised principal component analysis (PCA) was performed to test the population distribution of the samples and the stability of the whole analysis process, and the supervised (orthogonal) partial least squares discriminant analysis (OPLS-DA) was carried out to discriminate between metabolic profiles of the general differences between groups, and identify the differences between groups of metabolites. In OPLS-DA analysis, a variable important in projection (VIP) with value greater than 1 is considered as a differential variable. In order to prevent overfitting of the model, seven cyclic interactive validation and 200 response sequencing tests were used to assess the quality of the model.

### 4.6. HPLC of Flavonoid Aglycones

Mature leaves were collected from the 1-month-old pot-grown plants, immediately ground in liquid nitrogen with a mortar and pestle, transferred into an Eppendorf tube and weighed. For each genotype, mature leaves from three individual plants were used for flavonoid aglycones extraction. The extraction experiments were conducted with three biological replicates.For determination of total flavonoids, 100 mg fresh samples were extracted with 1 mL of 80% methanol, sonicated for 1 h and kept at room temperature on a rotating wheel overnight. The extract was centrifuged at room temperature for 30 min to remove tissue debris and the supernatant was dried under nitrogen gas, and the remnant was deglycosylated by redissolving the pellets in 2 M HCl and heated at 80 ℃ for 1 h. Flavonoid aglycones were extracted by shaking in an equal volume of ethyl acetate, and the ethyl acetate fraction was evaporated in a speed-vac centrifuge. The pellets were redissolved in an equal volume of 100% HPLC-grade acetone. Flavonoids were separated on an Agilent 6470 Triple Quadrupole LC/MS (QQQ LC/MS) system (Agilent Technologies, Santa Clara, CA, USA) equipped with an ions trap (IT) mass spectrometer. Solvent A was Milli-Q purified water, and solvent B was 100% methanol: 0–1 min 30% B, 1–6 min 90% B, 6–13 min 90% B, 13–20 min 50% B, 20–23 min 20% B, 23–25 min 20% B. The flow rate was 0.5ml/min. For determination of the relative abundance of the compounds, peak areas were integrated using the software implemented in the QQQ LC/MS system. To identify the separated peaks, a number of standard flavonoids and precursors, dissolved at 1, 0.5, 0.25, 0.125 and 0.0625 ppm, were separated under the same conditions, including naringenin, apigenin, quercitrin, liquiritigenin, isoliquiritigenin, dihydromyricetin, dihydroquercitrin, kaempferol and myricetin. HPLC analysis of flavonoid extracts was performed three times using different batches of transformed plants. All batches yielded similar results.

### 4.7. GA Measurements

The fresh leaves of 1-month-old soil-grown *ga3ox1* and R108 plants were collected and frozen in liquid nitrogen immediately. GAs was purified and quantified according to previous publication [93] by liquid chromatography-electrospray ionization tandem mass spectroscopy (LC-ESI-MS/MS) using an Xciex QTRAP 5500 rapid resolution LC-MS/MS system equipped with Turbo V source ESI probe (AB Xciex LLC, Ontario, Canada) fitted with an HSS T3 column (1.8 μm, 100 × 2.1 mm). The inlet method was set as follows: Solvent A (0.1% formic-aqueous solution), solvent B (0.1% formic-acetonitrile). Gradient: 0–17 min, 3% B to 65% B; 17–18.5 min, 65% B to 90% B; 18.5–19.5 min, 90% B; 19.5–21 min, 90% B to 3% B; 21–22.5 min, 3% B. GAs were detected in negative multiple reaction monitoring (MRM) mode. The source parameters were set as: IS voltage −4500 V, TEM 550°C, GS1 50, GS2 50 and curtain gas 40. Using the default parameters, the ion fragments were automatically identified and integrated in the Analyst Software (AB Sciex LLC). Taking the mass spectral peak area of the analyte as the vertical coordinate and the concentration of the analyte as the horizontal coordinate, the linear regression standard curve was drawn to calculate the sample concentration, and the mass spectral peak area of the sample analyte was substituted into the linear equation to calculate the concentration. To identify the separated peaks, a number of GAs, dissolved at 1, 5, 10, 20, 40, 60, 80, 120, 160, 200 ng/mL, were separated under the same conditions, including GA_1_ (CAS: 545-97-1, OlChemIm, Olomouc, Czech Republic), GA_3_ (CAS: 77-06-5, OlChemIm, Olomouc, Czech Republic), GA_4_ (CAS: 468-44-0, OlChemIm, Olomouc, Czech Republic), GA_7_ (CAS: 510-75-8, OlChemIm, Olomouc, Czech Republic). HPLC analysis of GA extract was performed three times using different batches. All batches yielded similar results.

### 4.8. Cell Area Measurement by Scanning Electron Microscopy (SEM)

The petioles and mature leaves of 1-month-old hydroponic-grown *ga3ox1* and R108 plants were harvested and fixed with a 3% glutaraldehyde solution (*v*/*v* in 1 × PBS) and 1% OsO_4_ (*v/v* in H_2_O) and dehydrated with a graded ethanol series (30, 50, 60, 70, 80, 90, 95 and 100%). Before SEM observations, tissues were critical-point dried in liquid CO_2_, mounted on aluminum stubs, dissected and sputter-coated with gold. Specimens were then examined using a ZEISS DSM-960A scanning electron microscope (Carl Zeiss, Microscopy GmbH, Oberkochen, Germany) at an accelerating voltage of 5 kV. Digital images were collected and assembled using Adobe Photoshop (Adobe Inc., San Jose, CA, USA).

### 4.9. qRT–PCR Analysis

Total RNA was extracted from 4-week-old *M. truncatula* leaves under hydroponic conditions using the Eastep^TM^ Super Total RNA Extraction Kit (Cat# Z3101; Promega Corporation, Madison, WI, USA) according to the manufacturer’s protocol. Full-length cDNA was then reverse transcribed using the cDNA synthesis kit PrimeScript RT Reagent Kit with gDNA Eraser (Cat# RR047A, TaKaRa, Tokyo, Japan). The qRT-PCR analysis was performed using a one-step qRT-PCR kit (Cat# RR420A; TaKaRa) according to the manufacturer’s instructions, using three independent RNA preparations as biological replicates. Actin gene transcript was used as a reference. The relative quantification (2^−△△CT^) of gene expression was evaluated using the comparative cycle threshold method [94]. The relevant primer sequences are given in Supplementary Materials Tables S6–S8.

### 4.10. Exogenous GA Treatment

After germination of wild-type R108 and *ga3ox1* mutant seeds, 7-day-old seedlings were selected and transplanted to 1/2 Hoagland nutrient solutions containing GA at the final concentrations of 10 μM (GA_1_, GA_3_, GA_4_ or GA_7_), and 100 μM prohexadione calcium, which were replaced once a week, respectively. Samples were collected from different organs of 4-week-old *M. truncatula* plants and frozen in liquid nitrogen immediately.

### 4.11. Determination of the Content of Chlorophyll, Proanthocyanidin and Flavonoids

The mature leaves of 1-month-old hydroponic-grown *ga3ox1* and R108 plants were obtained, and the chlorophyll content was determined according to a previously published study [95]. The content of proanthocyanidin and flavonoids were determined with a Proanthocyanidin Kit (Cat# BC1350; Solarbio Life Sciences, Beijing, China) and a Flavonoids Kit (Cat# BC1330; Solarbio Life Sciences, Beijing, China) following the manufacturer’s instructions.

### 4.12. Determination of Nitrate Reductase and Glutamine Synthase Activities

Glutamine synthase and nitrate reductase activities were determined in vitro using a Glutamine Synthase Kit (Cat# BC0910; Solarbio Life Sciences, Beijing, China) and the Nitrate Reductase Kit (Cat# BC0080 Solarbio Life Sciences, Beijing, China) following the manufacturer’s instructions.

### 4.13. Inoculation of Symbiotic Bacteria

For nodulation experiments in perlite, 2-day-old seedlings were transferred to perlite and grown for 5 days in nitrogen-poor medium before inoculation. The nodule number was counted at 14 dpi. At 14 days post-inoculation (dpi), nodule numbers were counted and main root lengths were measured with ImageJ software. Nodules appeared on both the main root and on some lateral roots; all nodules were taken into account for the analysis.

## 5. Conclusions

In summary, GAs, positive regulators of nitrogen metabolism, as well as negative regulators of flavonoid biosynthesis, play an essential role in balancing the relationship between flavonoid biosynthesis and nitrogen metabolism. DELLA proteins accumulation (due to GA deficiency) inhibited the expression of genes involved in nitrogen metabolism and stimulated the expression of genes involved in flavonoid biosynthesis.

## Data Availability

The mass spectrometry proteomics data have been deposited to the ProteomeXchange Consortium (http://proteomecentral.proteomexchange.org, 31 May 2021) via the iProX partner repository with the dataset identifier PXD026363 [96].

## Supplementary Materials

The following are available online at https://www.mdpi.com/article/10.3390/ijms22179291/s1.

## Abbreviations

GA: gibberellin acid; 2ODD: 2-oxoglutarate-dependent dioxygenase; ABA: abscisic acid; IAA: indole-3-acetic acid; SA: salicylic acid; ProCa: prohexadione calcium; PAL: phenylalanine ammonialyase; CHS: chalcone and stilbene synthase family protein; CHI: chalcone-flavanone isomerase family protein; ANR: anthocyanidin reductase; F3′H: flavonol synthase/flavanone 3-hydroxylase; LDOX: leucoanthocyanidin dioxygenase-like protein; DFR: dihydroflavonol 4-reductase-like protein; FLS: cytochrome P450 family flavone synthase; IOMT: Isoflavone-7-O-methyltransferase; AACT: anthocyanin 5-aromatic acyltransferase; PAP1: production of anthocyanin pigment 1; PAP2: production of anthocyanin pigment 2; GS: glutamine synthase; NR: nitrate reductase; GLN: Glutamyl-tRNA (Gln) amidotransferase; AAT: amino acid transporter; NRT: nitrate transporter; AMT: ammonium transporter; FNR: ferredoxin-nitrite reductase; PKL: CHD3-type chromatin-remodeling factor pickle protein; NCR: Nodule Cysteine-Rich (NCR) secreted peptide; Dof: DNA-binding with one finger; DEPs: differentially enriched proteins; KEGG: Kyoto Encyclopedia of Genes and Genomes.
