# Supplementary material for: Gibberellins Inhibit Flavonoid Biosynthesis and Promote Nitrogen Metabolism in Medicago truncatula
Source: Int J Mol Sci. 2021 Aug 27;22(17):9291. doi: 10.3390/ijms22179291 (PMC8431309; doi:10.3390/ijms22179291)
Supplement: Supplementary file 1 [file ijms-22-09291-s001.zip › Supplementary Files/Supplemental figures.pdf]

Supplemental figures

Supplementary Figure S1 Functional identification of MtGA3ox1 and profile of endogenous GAs in wild type R108 and *ga3ox1* mutant. **(A)** RT-PCR for *MtGA3ox1* in leaves of wild type R108 and three *ga3ox1* mutant lines. Actin was used as a reference gene. **(B)** Phylogenetic tree analysis. The phylogenetic tree was constructed by the neighbor-joining method with 1000 bootstrap replicates by using the software MEGA7.0 (http://www.megasoftware.net/). Numbers indicate confidence percentages. **(C)** GAs biosynthesis pathway. **(D)** Profile of endogenous GAs in wild type R108 and *ga3ox1* mutant based on HPLC-MS analysis. The GA levels were determined in leaves of one-month-old *M. truncatula*, which are presented as the mean ± SD (n=3 biological independent samples). *P<0.05, **P <0.01, and ***P <0.001.

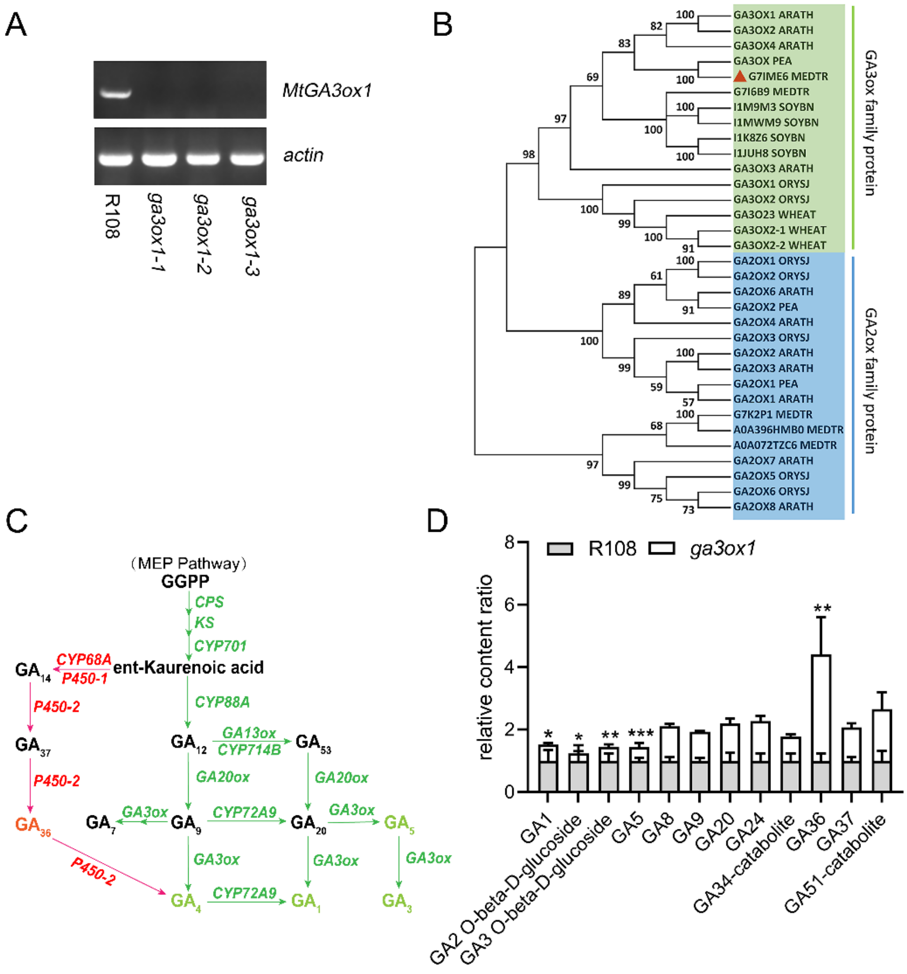

Supplementary Figure S2 Phenotypic analysis of leaf and seed coat color in *ga3ox1* mutant. **(A)** Phenotype of *ga3ox1* mutant leaf color. Bar = 1 cm. **(B)** Determination of chlorophyll content. Three biological replicates were analyzed. **(C)** Phenotype of *ga3ox1* mutant seed coat color. Bar = 0.5 cm. **(D)** Determination of flavonoid content in wild type R108 and *ga3ox1* mutant seeds. Three biological replicates were analyzed and error bars denote the SD. ** P < 0.01 by Duncan test.

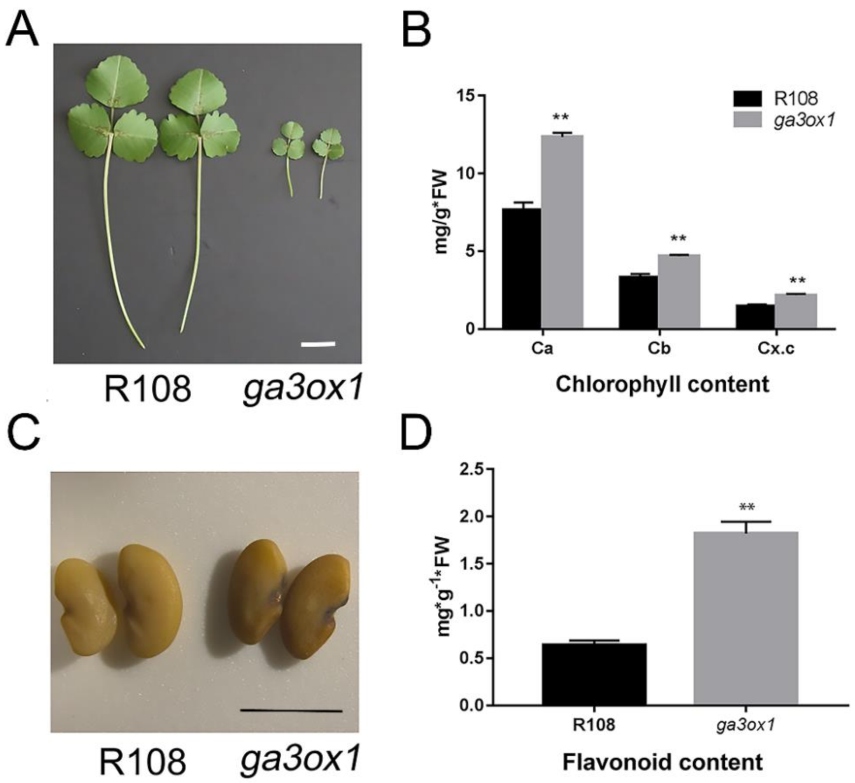

Supplementary Figure S3 Overexpression of *MtGA3ox1* rescues the dwarf phenotype in *ga3ox1* mutant. **(A)** Phenotype of the overexpression of *MtGA3ox1* in *ga3ox1* mutant. The *ga3ox1* mutant display a dwarf phenotype, whereas overexpression of *MtGA3ox1* rescues it. Bar = 2 cm. **(B)** Seed coat color was restored by the overexpression of *MtGA3ox1* in *ga3ox1* mutant. The *ga3ox1* mutant seed coat displays a dark brown color, whereas overexpression of *MtGA3ox1* restores it. Bar = 1 cm.

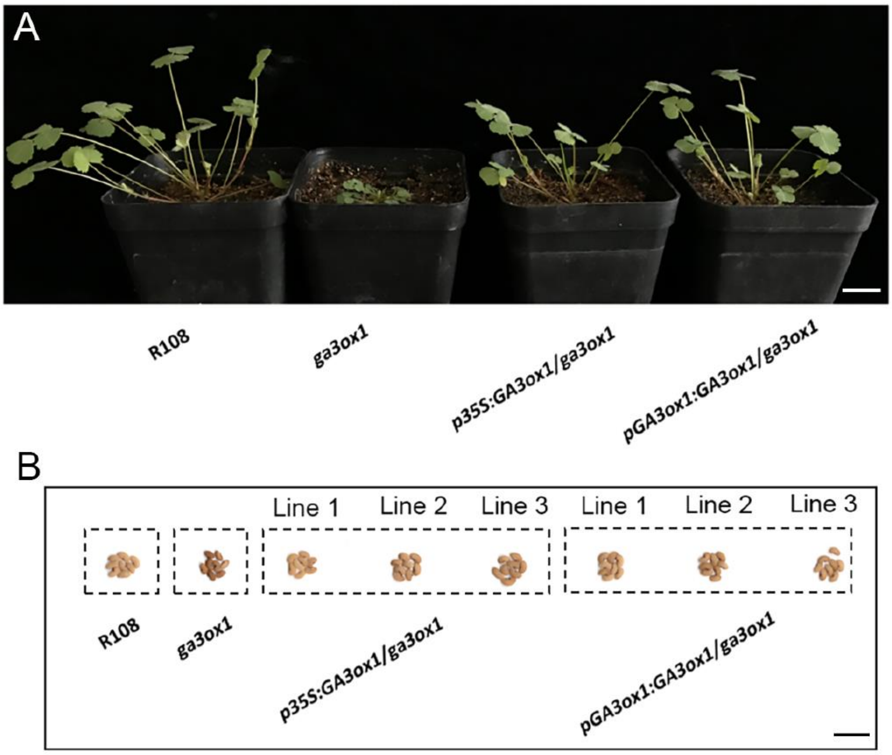

Supplementary Figure S4 Phenotype of wild type R108 and *ga3ox1* mutant treated with four bioactive gibberellins. **(A)** Phenotype of *ga3ox1* mutant under treatment with 10μmol/L GAs (GA_1, GA_3, GA_4, GA_7), and SEM images of leaves with a magnification of 500× and petioles with a magnification of 200×. **(B)** Leaf area of wild type R108 and *ga3ox1* mutant was measured with a leaf area meter. Six single leaves were randomly selected to calculate the cell area. **(C)** Leaf cell area of wild type R108 and *ga3ox1* mutant calculated using the ImageJ software. 10 single cells were randomly selected to calculate the leaf area. **(D)** Petiole length of wild type R108 and *ga3ox1* mutant were measured with a ruler. Six single petioles were randomly selected to calculated petiole length. **(E)** Petiole cell length of wild type R108 and *ga3ox1* mutant calculated using the ImageJ software. 10 single cells were randomly selected to calculate the petiole cell length. Error bars indicate the SD. * $P < 0.05$, and ** $P < 0.01$ by Duncan test.

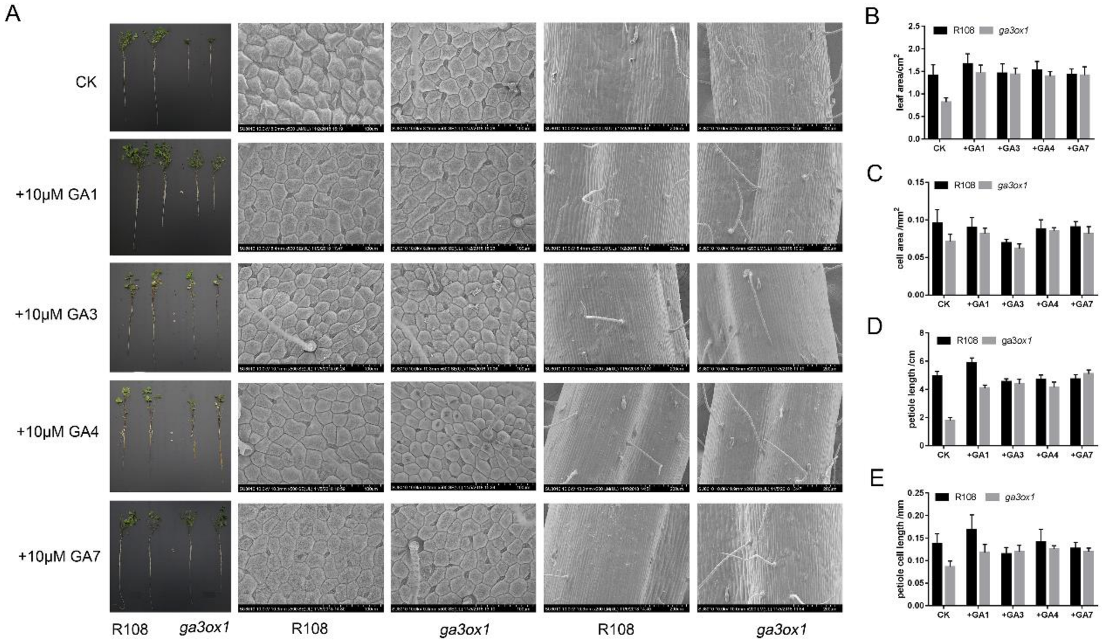

Supplementary Figure S5 The KEGG enrichment analysis of DEPs and differential metabolites. **(A-B)** KEGG enrichment analysis of upregulated, and downregulated gene expression. **(C)** Significant KEGG enrichment analysis of differential metabolites. The senior bubble was performed using the OmicShare tools, a free online platform for data analysis (http://www.omicshare.com/tools). According to the color scale on the right of the figure, each colorized cell represents the gene number, and the color scale indicates the *P*-value.

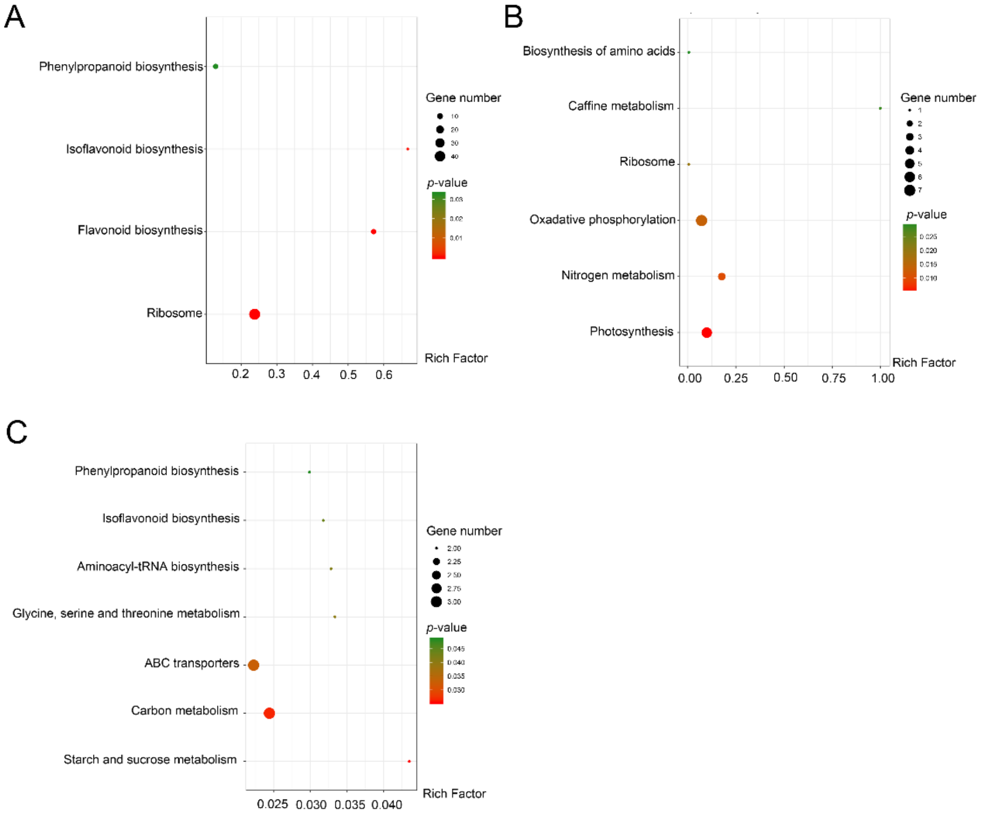

Supplementary Figure S6 The determination of flavonoid and proanthocyanin and qRT-PCR analysis to confirm the expression pattern of genes related to flavonoid biosynthesis. **(A-G)** Transcriptional expression levels of flavonoid biosynthesis genes determined in the *MtGA3ox1*-overexpression lines. **(H-I)** Flavonoid content and proanthocyanin content of leaves were detected by the spectrophotometry. Three biological replicates were analyzed and error bars denote the SD. DW: dry weight.

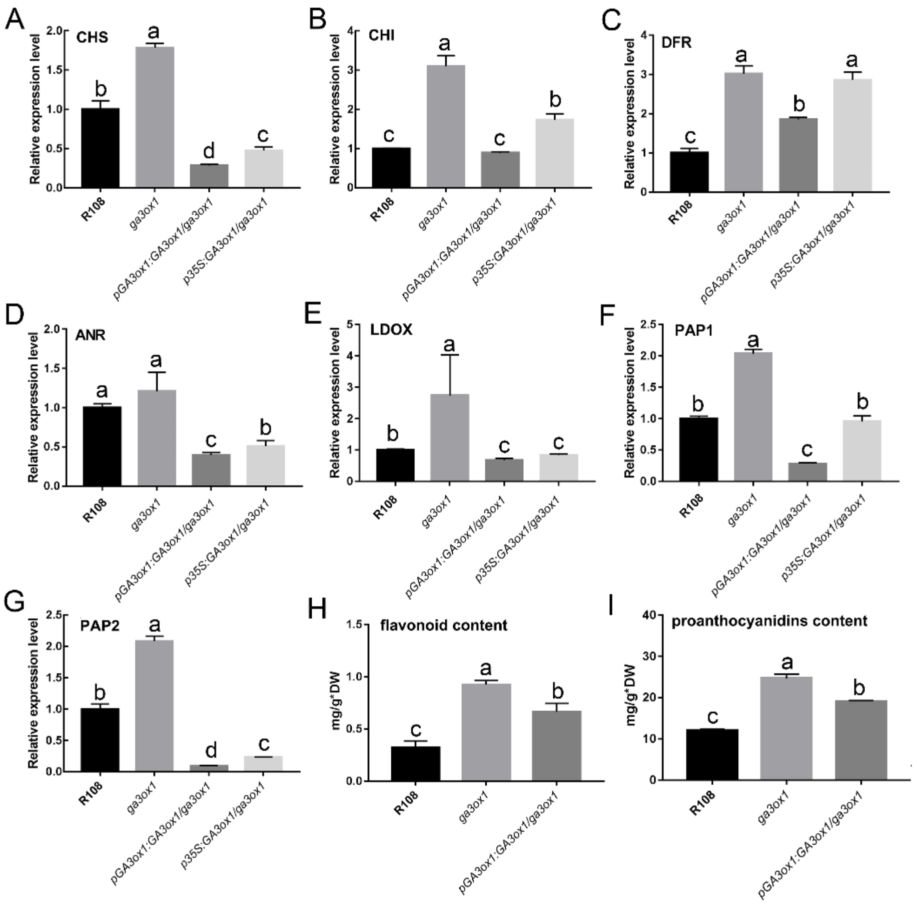

Supplementary Figure S7 HPLC chromatograms and absorbance spectra of standards. **(A)** HPLC chromatograms and absorbance spectra of standards mixture at the concentration of 1 ppm. **(B-J)** Retention times of myricetin, quercetin, apigenin, kaempferol, isoliquiritigenin, liquiritigenin, dihydromyricetin, dihydroquercetin, and naringenin standards.

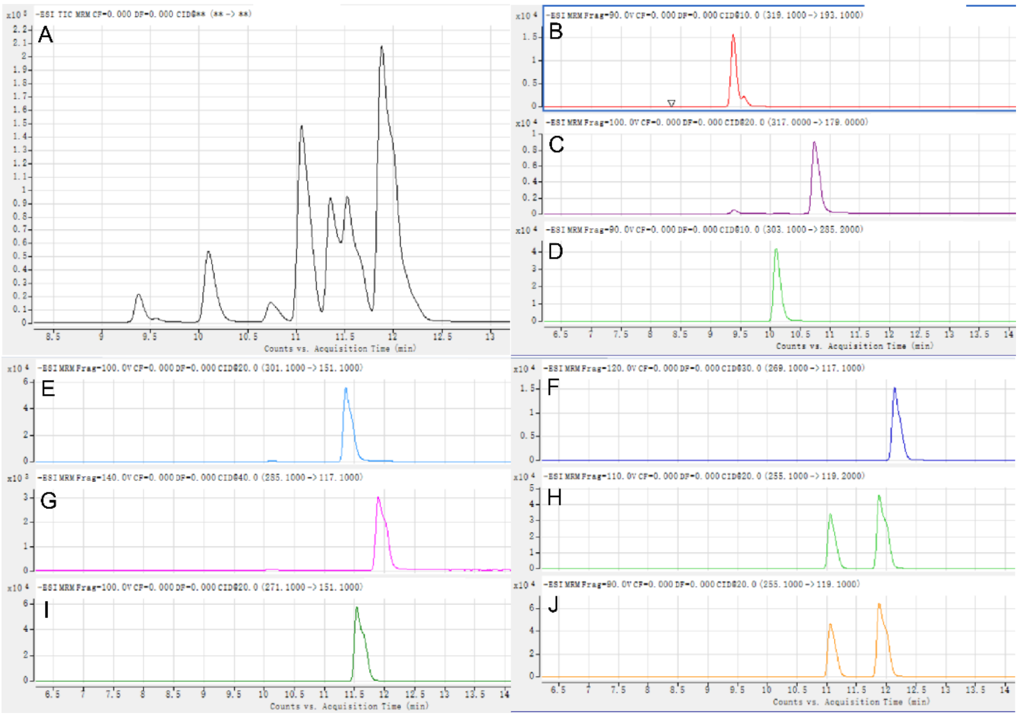

Supplementary Figure S8 qRT-PCR analysis to confirm the expression pattern of genes related to nitrogen metabolism and transport. **(A-D)** Transcriptional expression levels of several genes associated with nitrogen metabolism and transport were determined in *MtGA3ox1*-overexpression lines. **(E-F)** Glutamine synthase activity and nitrate reductase activity of leaves were detected by the spectrophotometry. Three biological replicates were analyzed and error bars denote the SD. FW: Fresh weight.

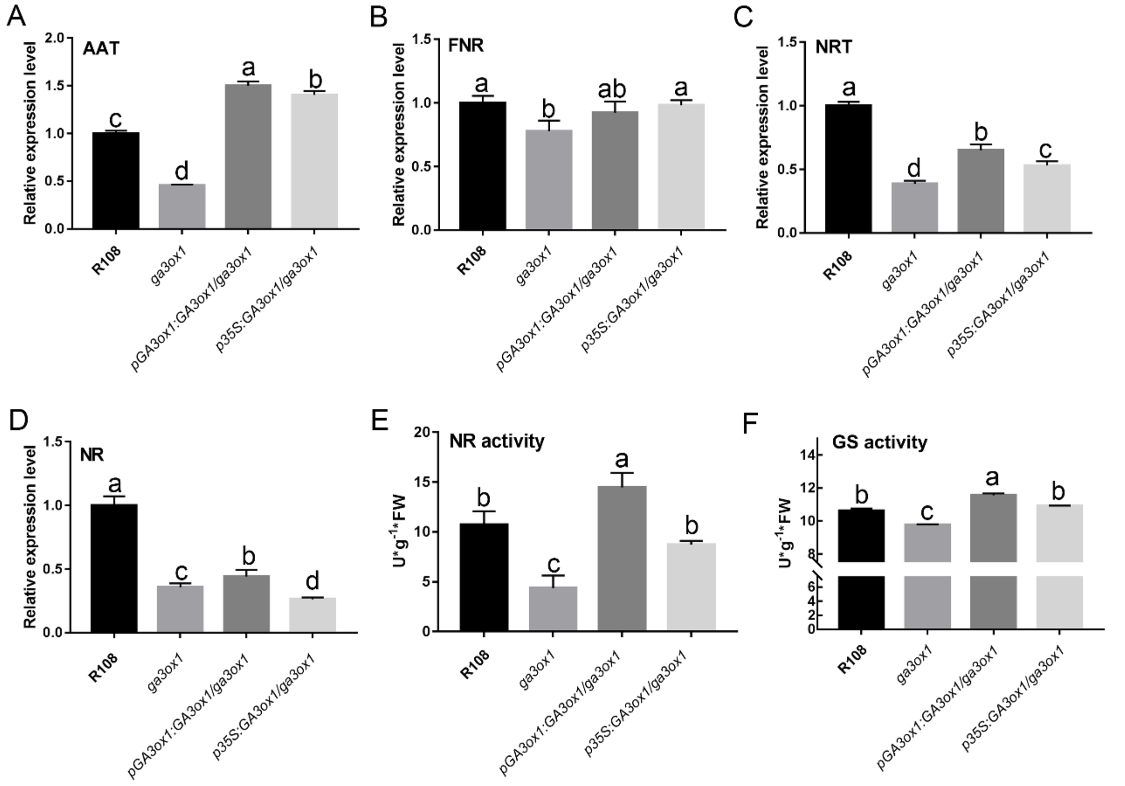

Supplementary Figure S9 Rhizobia inoculation and determination of DELLA protein and mRNA abundance. (A) The determination of DELLA protein and mRNA abundance. Three biological replicates were analyzed and error bars denote the SD. (B) Inoculation of symbiotic bacteria and Statistics of the calculation of average nodule number. Ten biological replicates were analyzed and error bars denote the SD. (C) The determination of root length and lateral root number. Ten biological replicates were analyzed and error bars denote the SD.

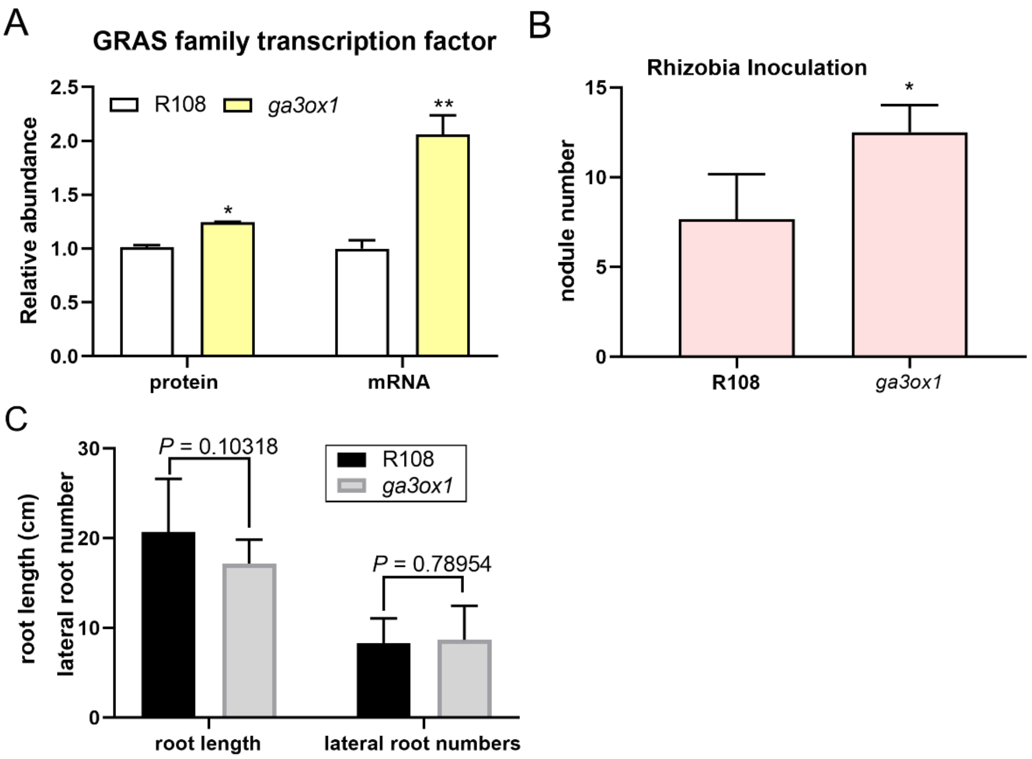

Supplemental Tables

Supplementary Table S1 The differentially enriched proteins identified by iTRAQ.

Supplementary table S2 The KEGG enrichment analysis of DEPs and differential metabolites.

Supplementary table S3 The differential metabolites identified by HPLC-MS.

Supplementary table S4 Primer for TAIL-PCR flanking sequence amplification.

Supplementary table S5 Primers for gene cloning and qRT-PCR.

Supplementary table S6 Quantitative primers for proteomics validation.

Supplementary table S7 Quantitative primers of genes related to nitrogen metabolism.

Supplementary table S8 Quantitative primers of genes involved in flavonoid and isoflavonoid biosynthesis.
